# Immunogenicity and safety of COVID-19 BNT162b2 booster vaccine in end-stage kidney disease patients receiving haemodialysis in Yogyakarta, Indonesia: a cohort prospective study

**DOI:** 10.1186/s12882-023-03218-x

**Published:** 2023-05-30

**Authors:** Metalia Puspitasari, Prenali D. Sattwika, Auliana R. P. Hidayat, Wynne Wijaya, Yulia Wardhani, Umi S. Intansari, Nyoman Kertia, Bambang Purwanto, Jarir At Thobari

**Affiliations:** 1 Department of Internal Medicine, Faculty of Medicine, Public Health and Nursing, Universitas Gadjah Mada/Dr. Sardjito General Hospital, Yogyakarta, Indonesia; 2 Clinical Epidemiology and Biostatistics Unit, Faculty of Medicine, Public Health and Nursing, Universitas Gadjah Mada/Dr. Sardjito General Hospital, Yogyakarta, Indonesia; 3 Cardiovascular Clinical Research Facility, Division of Cardiovascular Medicine, Radcliffe Department of Medicine, University of Oxford, Oxford, UK; 4 Department of Clinical Pathology and Laboratory Medicine, Faculty of Medicine, Public Health and Nursing, Universitas Gadjah Mada/Dr. Sardjito General Hospital, Yogyakarta, Indonesia; 5 Department of Internal Medicine, Faculty of Medicine, Universitas Sebelas Maret, Surakarta, Indonesia; 6 Department of Pharmacology and Therapy, Faculty of Medicine, Public Health and Nursing, Universitas Gadjah Mada, Yogyakarta, Indonesia

**Keywords:** COVID-19 vaccines, Booster, End-stage renal disease, Haemodialysis, Immunogenicity, Safety

## Abstract

**Background:**

A significant decrease in antibody titres several months after COVID-19 primary vaccination in end-stage kidney disease (ESKD) patients receiving maintenance haemodialysis has recently been reported. The waning in antibody titres has led to the recommendations for a booster dose to increase the antibody titres after vaccination. Consequently, it is crucial to analyse the long-term humoral immune responses after COVID-19 primary vaccination and assess the immunogenicity and safety of booster doses in haemodialysis (HD) patients.

**Methods:**

Patients on maintenance haemodialysis who received the primary vaccine of CoronaVac (Sinovac) vaccine were administered with BNT162b2 (Pfizer-BioNTech) as the booster dose. The immunogenicity was assessed before (V1), one month (V2) and eight months (V3) after the primary vaccination, as well as one month after the booster dose (V4). Patients were followed up one month after the booster dose to assess the adverse events (AEs).

**Results:**

The geometric mean titre (GMT) of anti-SARS-CoV-2 S-RBD IgG antibody at 8 months after the primary vaccination increased significantly to 5,296.63 (95%CI: 2,930.89–9,571.94) U/mL (*p* =  < 0.0001) compared to before the primary vaccination. The GMT also increased significantly to 19,142.56 (95% CI: 13,489.63–27,227.01) U/mL (*p* < 0.0001) 1 month after the booster vaccine. Meanwhile, the median inhibition rate of neutralizing antibodies (NAbs) at 8 months after the primary vaccine and 1 month after the booster dose were not significantly different (*p* > 0.9999). The most common AEs after the booster dose included mild pain at the injection site (55.26%), mild fatigue (10.53%), and swelling at the injection site (10.53%). No serious AEs were reported.

**Conclusions:**

The majority of ESKD patients on haemodialysis mounted a good antibody response to the BNT162b2 booster vaccination with tolerable adverse events.

**Supplementary Information:**

The online version contains supplementary material available at 10.1186/s12882-023-03218-x.

## Background

The coronavirus (COVID-19) pandemic has profoundly impacted all aspects of society and the health system. End-stage kidney disease (ESKD) patients undergoing haemodialysis have a higher risk of SARS-CoV-2 infection due to frequent healthcare contacts and a high burden of comorbid diseases [1]. Nowadays, effective vaccination has become a reliable means to reduce COVID-19 morbidity and mortality. The administration of COVID-19 vaccines to vulnerable populations such as ESKD patients is a priority, as recommended by the UK Renal Association and the US National Kidney Foundation [2]. The challenge after COVID-19 vaccination is in the longevity and kinetics of antibody response over the months following vaccination, particularly against variants of concern and in susceptible populations [3]. In addition, haemodialysis patients have been demonstrated to mount lower responses to vaccination [4].

Although various types of COVID-19 vaccines are known to have high effectiveness, reports regarding COVID-19 breakthrough infections in patients who have received two doses of the vaccine have already emerged [5–7]. Moreover, some studies in maintenance haemodialysis patients have demonstrated a significant decrease in antibody titres 6 months after the primary vaccination [8–10]. The waning in antibody titres has led to recommendations for the administration of a booster dose to sustain the antibody titres [11]. To our knowledge, studies regarding the immunogenicity of heterologous vaccines, with CoronaVac (Sinovac) as the primary vaccine and BNT162b2 as the booster dose in haemodialysis patients are still scarce. Moreover, there are currently no studies that have examined the immunogenicity and safety of COVID-19 booster doses among Indonesian haemodialysis patients. In this study, we aim to evaluate the humoral immune response at 8 months after CoronaVac (Sinovac) primary vaccination and assess the immunogenicity and safety of BNT162b2 booster dose among local haemodialysis patients in Yogyakarta, Indonesia.

## Methods

### Study design and participants

This study is an observational cohort prospective study conducted on haemodialysis patients at the Renal Unit of Dr Sardjito General Hospital, Yogyakarta, Indonesia. Measurement of the humoral response after vaccination was performed four times: before the primary dose (V1), one month after the primary dose (V2), 8 months after the primary dose (V3), and one month after the booster dose of COVID-19 vaccination (V4). The median interval between the second dose and the booster dose was 263 (262–269) days. We included ESKD patients undergoing maintenance haemodialysis, aged 18–59 years, who have received two doses of the CoronaVac vaccine and the booster dose of BNT162b2 (Pfizer-BioNTech). Patients who presented with an acute and unstable condition related to ESKD or other diseases, developed a systemic infection during the study, were on steroid or immunosuppressant therapy, or had received a previous COVID-19 booster vaccination were excluded from this study.

### Data collection

Data was collected through history taking, physical examination, laboratory examination, and evaluation of data in medical records. Samples were obtained through the consecutive sampling method: each patient who met the inclusion and exclusion criteria was included in the study until the minimum number of samples was met. The minimum sample size was determined using the software: Power and Sample Size Calculation program version 3.1.2 by William Dupont and Walton Plummer Jr. The means and standard deviations of anti-SARS-CoV-2-IgG titres are needed to calculate the minimum sample size. For the independent group, n1 = n2, α = 0.05 and the desired research power (1 – β) = 0.95. From a previous study by Shashar et al., the mean difference before and after the booster vaccine is 15,864.2 U/mL, and the standard deviation after the booster is 15,397.3 U/mL [12]. After calculation, the minimum sample size is 14 subjects.

### Antibody measurements

The humoral response was assessed by measuring anti-SARS-CoV-2 S-RBD IgG and anti-sRBD neutralizing antibodies (NAbs) in patients’ blood samples. Antibody measurement was performed four times: before the primary vaccination (V1), 1 month after the primary vaccination (V2), 8 months after the primary vaccine (V3), and 1 month after the booster dose (V4). Anti-SARS-CoV-2 IgG sRBD antibodies were measured using the Elecsys® Anti-SARS-CoV-2 S (Spike) assay (Roche Diagnostics, Germany). Blood samples were incubated with a mix of biotinylated and ruthenylated RBD antigens. Double-antigen sandwich immune complexes (DAGS) are formed in the presence of corresponding antibodies. After the addition of streptavidin-coated microparticles, the DAGS complexes bind to the solid phase via the interaction of biotin and streptavidin. The reagent mixture is transferred to the measuring cell, where the microparticles are magnetically captured onto the surface of the electrode. Unbound substances are subsequently removed. Electrochemiluminescence is then induced by applying a voltage and measured with a photomultiplier. The signal yield increases with the antibody titre. According to the manufacturer’s instructions, patients with an antibody level of ≥ 0.8 U/mL were considered reactive or seropositive [13].

Blood specimens were tested for NAbs against S-RBD using a surrogate viral neutralization test (sVNT) (cPass™, GenScript USA). The cPass SARS-CoV-2 Neutralization Antibody Detection Kit is a blocking Enzyme-Linked Immunosorbent Assay (ELISA) intended for the qualitative and semi-quantitative direct detection of total NAbs to SARS-CoV-2 in human serum and dipotassium EDTA plasma. cPass™ is intended for use as an aid in identifying individuals with an adaptive immune response to SARS-CoV-2, indicating recent or prior infection. cPass™ is only for use under the Food and Drug Administration’s Emergency Use Authorization. According to the manufacturer’s instructions. Horseradish peroxidase (HRP)-RBD is pre-incubated with test serum (1:10 diluted) for 1 h at 37 °C. After that, it is added onto the ELISA plate pre-coated with hACE2 (GenScript). The unbound HRP-RBD is washed off and bound RBD-ACE2 is detected colourimetrically. Circulating NAbs against SARS-CoV-2 competitively inhibit the RBD-ACE2 interaction. The percentage of inhibition is calculated by measuring the difference in the amount of labelled RBD between test versus control samples. The cut-off value for neutralizing antibodies is ≥ 30% signal inhibition. [14].

To assess humoral response after the booster dose, we calculated the ratio of the antibody titres at V4 to the titres at V3.

### Adverse events (AE)

The patients were monitored for 1 month after the booster vaccination. Once a week, the patients were enquired about the reactions that were elicited by the vaccine throughout the week. The adverse events are categorized into local and systemic reactions. AEs are classified into Grade 1 to 4: Grade 1 AE represented mild symptoms (does not interfere with activity), Grade 2 entailed moderate symptoms (interferes with activity), Grade 3 entailed severe symptoms (prevents daily activity) and Grade 4 involved an emergency department visit or hospitalization. The grades were established according to the Food and Drug Administration toxicity grading scale [15].

### Ethical considerations

Informed consent was obtained from all subjects and/or their legal guardian(s). All methods were carried out in accordance with relevant guidelines and regulations i.e., Declaration of Helsinki and Good Clinical Practice. All study protocols were approved by The Medical and Health Research Ethics Committee of the Faculty of Medicine, Public Health, and Nursing, Universitas Gadjah Mada, Yogyakarta, Indonesia (No. KE/FK/1005/EC/2022).

### Statistical analysis

Descriptive data were presented as mean and standard deviation (SD) or median and interquartile range (IQR) for continuous data or percentage and proportion for dichotomous data. Differences in demographic data and clinical characteristics were tested with a mean difference test or proportion test. The normality of data distribution was tested with the Shapiro–Wilk test. The differences of IgG sRBD geometric mean titres were tested with one-way repeated measures ANOVA. To assess the effect of COVID-19 vaccination on the inhibition rate of NAbs anti-sRBD antibodies, anti-SARS-CoV-2 IgG sRBD antibody titres, incidence and severity of adverse events, a chi-square test or Fisher's exact test was performed if the data were not normally distributed. The differences in NAbs inhibition rate between the were tested with the Friedman test. Bivariate analysis was performed on each confounding variable with the dependent variable. Statistical analysis was performed using SPSS Statistics version 25.0. Results are considered statistically significant if *p* < 0.05 with 95% confidence intervals (CI).

## Results

Among the 38 participants, the median age was 49 (IQR: 40–53.25) years old and 52.6% were male (Table 1). The median interval between the second dose and the booster vaccine was 263 (262–269) days. The booster vaccine was administrated 1–2 days after the antibody measurement at V3.

**Table 1:** Baseline characteristics of study participants

Characteristics	N	%
**Sex, n (%)**		
Male	20	52.6
Female	18	47.4
**Age, years**		
Median (Q1-Q3)	49 (40–53.25)	
Min–max	28–60	
**Previous COVID-19 infection**		
Before the primary vaccine	4	12.5
After the primary vaccine	4	12.5

### Humoral Response

The anti-sRBD IgG GMT was 2.98 (1.20–7.42) U/mL at baseline (V1) and 332.66 (197.24–561.05) U/mL at 1 month after the primary vaccination (V1). The titre increased significantly to 5,296.63 (2,930.89–9,571.94) U/mL at 8 months after the primary vaccine (V3). Meanwhile, the median percentage of inhibition of NAbs was 15% (9–83.75%) at baseline (V1) and 84% (70.50–96%) 1 month after the primary vaccination (V2). Next, it increased significantly to 97% (93.50–98%) at 8 months after the primary vaccine (V3) (Table 2).

**Table 2:** Humoral immune response after vaccination at V1, V2, V3, and V4

Humoral immune response	**Value**	*p*-value^*^
**Anti-sRBD IgG antibody (U/mL), GMT (95%CI)**		
Baseline (V1)	2.98 (1.20–7.42)	Ref
1 month after primary vaccination (V2)	332.66 (197.24–561.05)	V1 vs V2: < 0.0001
8 months after primary vaccination (V3)	5,296.63 (2,930.89–9,571.94)	V1 vs V3: < 0.0001 V2 vs V3: < 0.0001
1 month after the booster (V4)	19,142.56 (13,489.63–27,227.01)	V1 vs V4: < 0.0001 V2 vs V4: < 0.0001 V3 vs V4: < 0.0001
**Anti-sRBD IgG antibody (U/mL), GMFR (95%CI)**		
ΔGMFR V2 vs. V1	111.69 (26.42–145.55)	NA
GMFR V3 vs. V1	1,782.38 (2.19–158,854.68)	NA
GMFR V4 vs. V1	6,426.88 (1,256.03–11,428.78)	NA
**sVNT NAbs (% inhibition), Median (IQR)**		
Baseline (V1)	15 (9–83.75)	Ref
1 month after primary vaccination (V2)	84 (70.50–96)	V1 vs V2: 0.0308
8 months after primary vaccination (V3)	97 (93.50–98)	V1 vs V3: < 0.0001 V2 vs V3: 0.0044
1 month after the booster (V4)	97 (96.75–98)	V1 vs V4: < 0.0001 V2 vs V4: 0.0007 V3 vs V4: > 0.9999

^*^Statistically significant if *p* < 0.05

The IgG sRBD GMT increased significantly to 19,142.56 (13,489.63–27,227.01) U/mL at V4 compared to V3 (*p* < 0.0001). Meanwhile, the median NAb percentages of inhibition at V3 and V4 were not significantly different (*p* > 0.9999) (Table 2).

Subjects were divided into two groups based on the IgG sRBD antibodies before and after the booster dose: increased and decreased IgG-sRBD antibody titre. Twenty-three subjects (73.68%) had increased IgG sRBD titres after the booster dose, while 10 (26.32%) subjects had decreased IgG sRBD titres after the booster dose (Table 3).

**Table 3:** Characteristics of subjects based on antibody increase after booster dose

Characteristics	Ratio of IgG sRBD at V4 to V3		*p* value^*^
	**Increase (*n* = 28)**	**Decrease (*n* = 10)**	
**Clinical characteristics**			
Sex, n (%)			
Male	14 (50%)	6 (60%)	0.587
Female	14 (50%)	4 (40%)	
Age, years	45 (40–52.7)	53 (46.5–54)	0.104
Haemodialysis adequacy (Kt/V)	1.71 ± 0.40	1.77 ± 0,35	0.748
**Laboratory characteristics**			
Hemoglobin, g/dL			
Mean ± SD	9.16 ± 1.43	9.17 ± 1.42	0.991
Lymphocyte count, 10^9^ cells/L			
Median (IQR)	1,485 (1,085–1,767.5)	1,215 (1,030–1,577.5)	
Thrombocyte count, 10^9^ cells/L			
Mean ± SD	215,392.86 ± 62,778.47	243,800.00 ± 79,333.05	0.259
BUN, mg/dL			
Mean ± SD	59.56 ± 12.98	57.04 ± 17.40	0.634
Creatinine, mg/dL			
Mean ± SD	12.17 ± 3.47	12.50 ± 4.12	0.804
Ferritin, ng/mL			
Median (IQR)	390.50 (217.25–800.00)	522.00 (140.00–741.45)	0.895
Albumin, g/dL			
Median (IQR)	4.10 (3.93–4.25)	4.00 (3.74 – 4.18)	0.246
**Humoral responses**			
**IgG-sRBD, U/mL**			
Baseline			
< 0.8 (Seronegative)	0	0	NA
≥ 0.8 (Seropositive)	6,231.50 (996.25–12,465.25)	22,085.50 (8,933.25–69,275.75)	0.004
28 days after booster dose	23,756.50 (12,517.50–41,339.25)	16,611.50 (7,872.50- 41,711.75)	0.507
Mean fold increase	4.23 (2.08–15.52)	0.71 (0.65–0.88)	0.000
**NAbs, % inhibition**			
Baseline			
< 30 (Seronegative)	0	0	NA
≥ 30 (Seropositive)	97.00 (89.75–97.00)	97.50 (94.75–98.00)	0.248
28 days after booster dose	97 (97–98)	96.5 (95–97.25)	0.123
Mean increase	1 (1–1.04)	1.00 (0.99–1.00)	0.041

^*^Statistically significant if *p* < 0.05

Subjects with increasing antibody titres after the booster dose were younger compared to those with decreasing antibody titres, but the difference is not statistically significant (*p* = 0.104). There was no significant difference in the haemodialysis adequacy based on Kt/V between the two groups and Kt/V was inadequate in both groups i.e., less than 1.8. The baseline laboratory characteristics are compared between the two groups in Table 3. Laboratory values for haemoglobin, lymphocyte count, platelet count, BUN, creatinine, ferritin, and albumin did not differ significantly between the two groups.

The NAbs percentage of inhibition and IgG sRBD antibodies showed seropositive results in all subjects before the administration of the booster dose. The IgG sRBD antibody titre before the booster dose was significantly higher in the group of subjects who had decreasing antibody titres after the booster (*p* = 0.004). However, the IgG sRBD antibody titres after the booster vaccine were not significantly different between the two groups. The NAbs percentage of inhibition before and after the booster dose was not significantly different between the two groups (*p* = 0.248, *p* = 0.123). However, the NAbs percentage of inhibition mean increase was significantly higher in the group with increasing IgG sRBD antibodies after booster (*p* = 0.041).

**Fig. 1:**
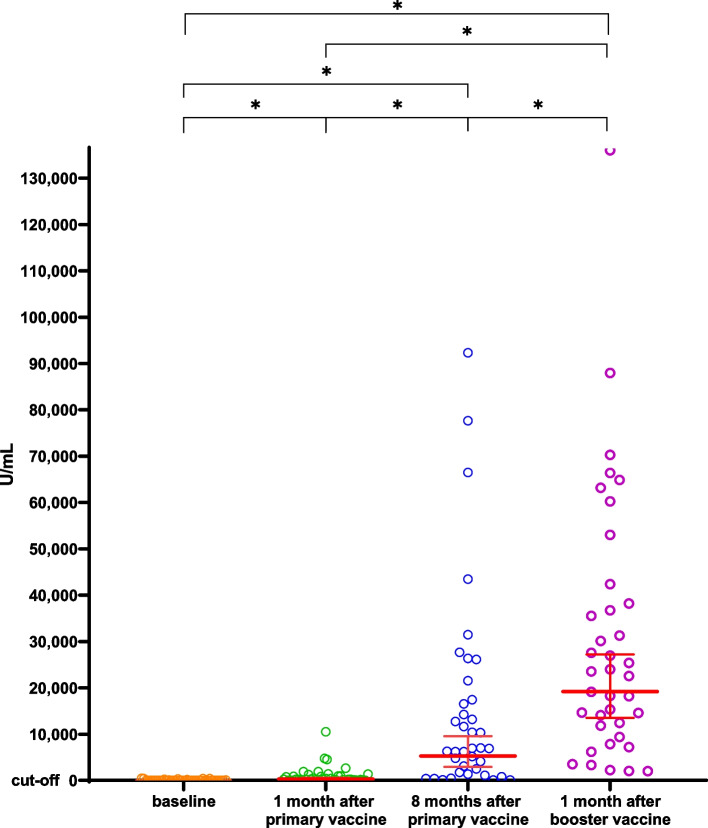
Scatter plot showing anti-sRBD IgG titre at V1, V2, V3, and V4 (X: sampling time; Y: IgG sRBD titre)

**Fig. 2:**
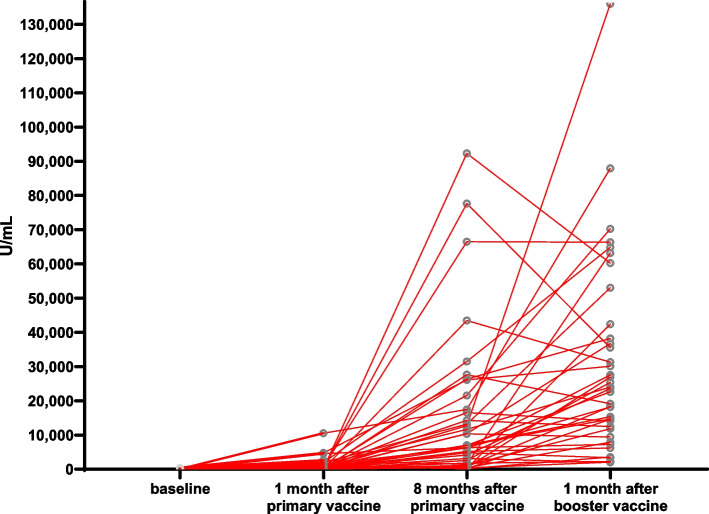
Change in anti-sRBD IgG titre at V1, V2, V3, and V4 (X: sampling time; Y: IgG sRBD titre)

**Fig. 3:**
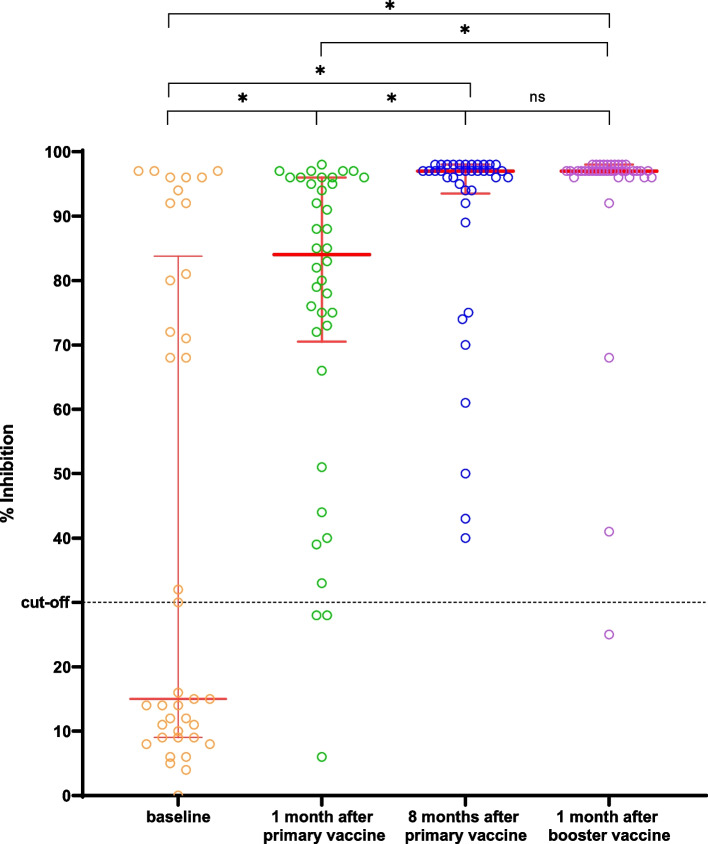
Scatter plot showing NAbs % inhibition at V1, V2, V3, and V4 (X: sampling time; Y: NAbs %inhibition)

**Fig. 4:**
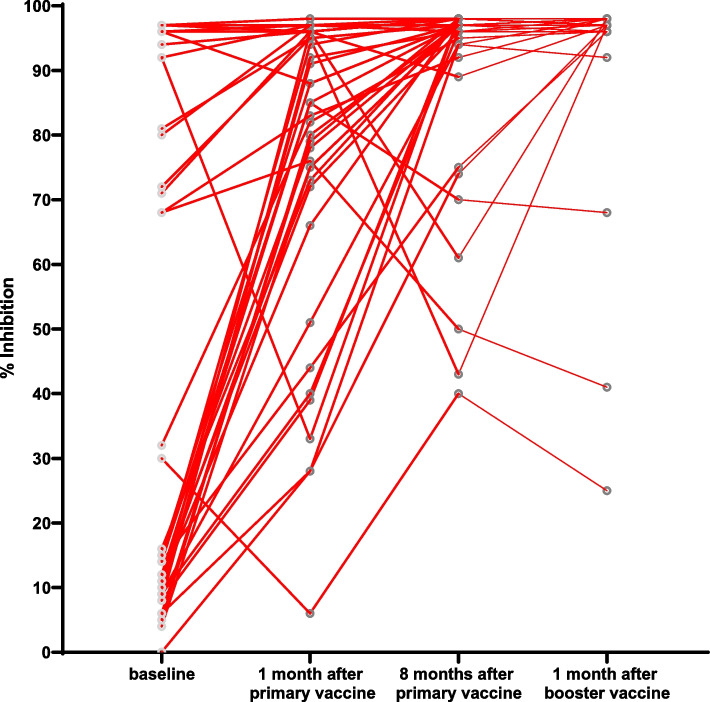
Change in anti-sRBD IgG titre at V1, V2, V3, and V4 (X: sampling time; Y: NAbs %inhibition)

Figure 1 shows the scatter plot of sRBD IgG levels through four points of time, whereas Fig. 2 demonstrates the change of sRBD IgG levels before the primary vaccination until 1 month after the booster dose vaccination. Figure 3 shows the scatter plot of the inhibition percentage of NAbs through four points in time. Figure 4 represents the change in the inhibition percentage of NAbs before the primary vaccination until 1 month after the booster dose vaccination. It was found that 1 study subject had extremely high post-booster vaccination IgG sRBD levels.

**Fig. 5:**
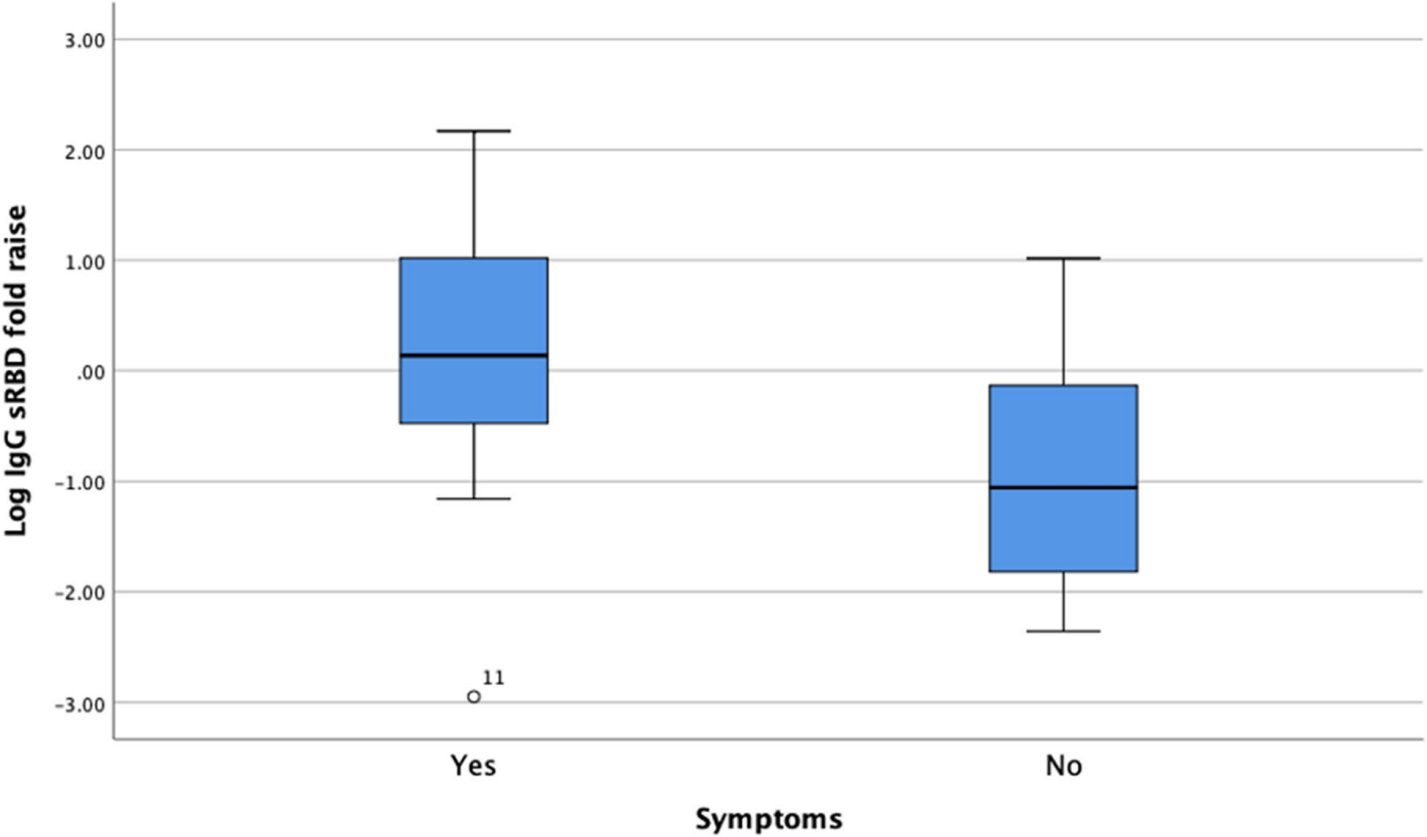
IgG sRBD ratio of V4 to V3 of symptomatic and asymptomatic haemodialysis patients

Furthermore, we conducted a separate analysis comparing the humoral response of patients who experienced COVID-19 symptoms and the asymptomatic ones (Table 4). Symptoms were traced from after the second vaccination until right before the booster dose administration. Interestingly, we found a significant difference in the IgG sRBD ratio of V4 to V3 between symptomatic (1.45 U/mL [IQR 0.27–11.74]) and asymptomatic patients (0.12 U/mL [IQR 0.02–0.05]) (*p* = 0.006) (Fig. 5).

**Table 4:** Humoral response of subjects based on the presence of COVID-19 symptoms before booster dose administration

Humoral response	Symptomatic (*n* = 18)	Asymptomatic (*n* = 20)	*p* value
Increasing IgG sRBD	16 (88.9%)	16 (80.0%)	0.663
Increasing NAbs	16 (88.9%)	16 (80.0%)	0.663
IgG sRBD Ratio V4 to V3	1.45 (IQR 0.27–11.74) U/mL	0.12 (IQR 0.02–0.05) U/mL	**0.006**

### Adverse Events (AE)

Five (13.16%) subjects experienced at least one solicited AE with 5 types of reactions in the first 30 min. On the first day, nineteen (50%) subjects experienced at least one solicited AE with 26 types of reactions. From day 2 to day 7, there were 12 (31.58%) subjects who experienced at least one solicited AE with a total of 17 types of reactions, among which one AE had a grade 3 severity. From day 2 to 7, one subject experienced an unsolicited AE of a grade 3 severity. From day 8 to 14, three subjects (7.89%) experienced at least one unsolicited AE (Additional file 1). The most common adverse events after the booster dose include mild pain at the injection site (55.26%), mild fatigue (10.53%), and swelling at the injection site (10.53%). No serious AEs were reported among the subjects after the booster dose (Additional file 2).

**Fig. 6:**
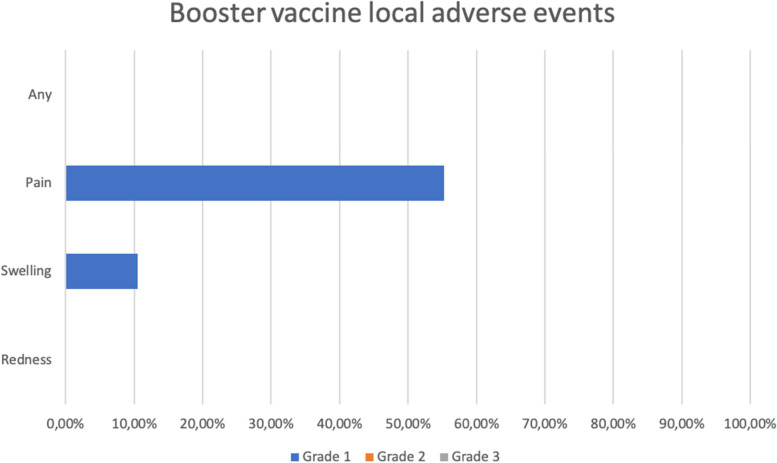
Local adverse events after the booster dose

**Fig. 7:**
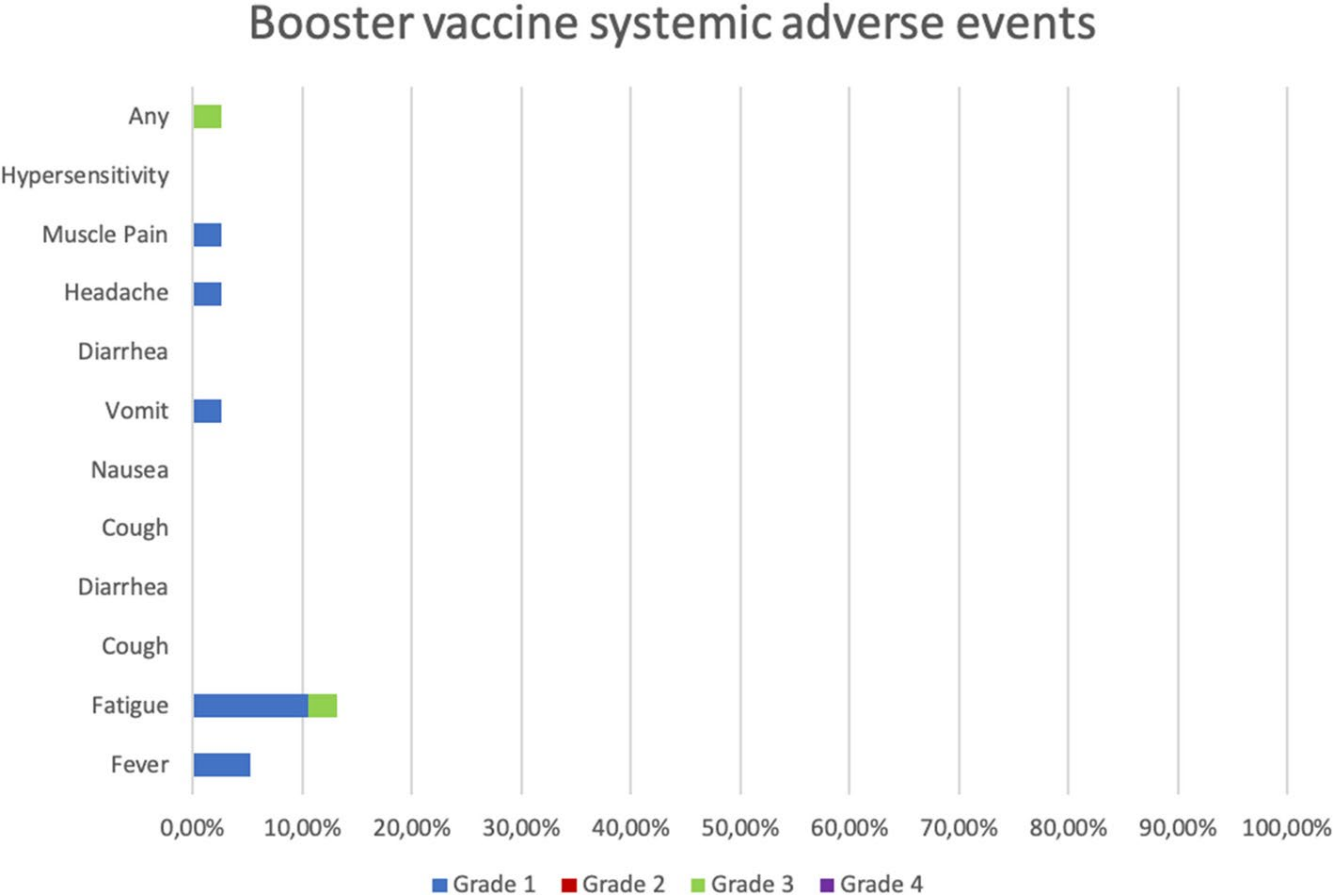
Systemic adverse events after the booster dose

Figure 6 demonstrates the most common local AEs after the booster dose, including pain (55.26%) and swelling (10.53%) at the injection site. Figure 7 shows that the most common systemic AEs after the booster dose were fatigue (13.26%), fever (5.26%), muscle pain (2.63%), headache (2.63%), and vomiting (2.63%).

Table 5 shows that the frequency of solicited AEs did not differ significantly based on co-morbidities, such as diabetes mellitus, hypertension, anaemia, and joint pain. However, the frequency of AEs was significantly higher in subjects with lower IgG sRBD antibody titres (*p* = 0.024).

**Table 5:** Solicited adverse events after booster vaccines based on characteristics

Characteristics	Solicited adverse events	No solicited adverse events	*p* value^*^
	**(*n* = 24)**	**(*n* = 14)**	
**Diabetes Mellitus**	2 (8.33%)	3 (21.43%)	0.249
**Hypertension**	21 (87.5%)	11 (78.57%)	0.467
**Anemia**	13 (54.17%)	8 (57.14%)	0.859
**Joint pain**	5 (20.83%)	4 (28.57%)	0.588
**Hemoglobin, g/dL**			
Mean ± SD	9.07 ± 1,29	9.34 ± 1,63	0.5777
**Lymphocyte, 10**^**9**^** cells/L**			
Median (Q1-Q3)	1475 (1025–1820)	1335 (1112,5–1577.5)	0.515
**Thrombocyte count/L**			
Mean ± SD	224,875.00 ± 74,217.84	219,428.57 ± 56,871.94	0.814
**BUN, mg/dL**			
Mean ± SD	57.91 ± 12.18	60.58 ± 17.20	0.58
**Creatinine, mg/dL**			
Mean ± SD	12.08 ± 3.34	12.56 ± 4.11	0.693
**Ferritin, ng/mL**			
Median (Q1-Q3)	464.50 (232.25–840.50)	367.50 (157.50–733.25)	0.477
**Albumin, g/dL**			
Median (Q1-Q3)	4.04 (3.94–4.23)	4.07 (3.85–4.26)	0.844
**Anti sRBD IgG, U/L**			
Median (Q1-Q3)	1561.5 (250–6,821.5)	2500 (250–6,436)	**0.024**
**NAbs, % inhibition**			
Median (Q1-Q3)	97 (94.5–98)	96.5 (74.75–98)	0.404

^*^Statistically significant if *p* < 0.05 (typed in bold)

## Discussion

This study assessed the humoral response to COVID-19 vaccination in HD patients by measuring the IgG sRBD titres and the NAbs percentage of inhibition at four points of time: before vaccination (V1), 1 month after the primary COVID-19 vaccination (V2), 8 months after the primary vaccination (V3), and 1 month after the booster dose (V4).

At 8 months after the primary vaccine (V3), the median NAbs percentage of inhibition and the IgG sRBD antibodies titre increased significantly compared to 1 month after the primary vaccine (V2). One possible explanation is the exposure to COVID-19 infection. However, this hypothesis cannot be proven since we did not measure the antibodies to nucleocapsid (anti-N IgG) in this study. Another plausible hypothesis is that the humoral response might just have been maintained for months after the primary vaccination without any new exposure or infection of COVID-19. Among subjects with increasing NAbs percentage of inhibition and IgG sRBD antibodies titre from V2 to V3, four (12.5%) subjects were confirmed positive for COVID-19 with RT-PCR, and 12 (37.5%) experienced COVID-19 symptoms but did not get tested. This finding is consistent with a previous study by Zhong et al., which demonstrated that vaccinated subjects with previous COVID-19 infection had a higher increase in antibody levels compared to individuals without previously confirmed COVID-19 infection [16]. Another study by Fuëssl et al. revealed a similar finding i.e., previous COVID-19 infection was associated with higher antibody response after the first booster vaccination (*p* = 0.001) [17]. A systematic review and meta-analysis also demonstrated a stronger humoral response in haemodialysis patients with previous COVID-19 infection compared to those without. In this study, Peiyao et al. showed that the seropositive conversion rate in patients without prior infection (82.9%) was significantly lower than in patients with prior infection (98.4%) (p < 0.00001). In addition, when the antibody titres were compared among the two groups, the mean difference was 1.14, indicating that patients with prior infection are more likely to develop antibodies [18]. Not only in haemodialysis patients, this pattern of response has also been demonstrated in the general population. Prendecki et al. found that anti-S titres were significantly higher in individuals with previous natural infection (16,535 AU/mL [IQR 4,741–28,581]) than in infection-naïve individuals (615.1 AU/mL [IQR 286.4–1,491]; *p* < 0.0001) [19].

Sixteen (50%) subjects with increasing antibody titres at V3 never experienced any symptoms of COVID-19. This might imply that primary vaccination can reduce the severity of COVID-19 infection. Similarly, a previous study by Ranzani et al. also showed that the effectiveness of the Sinovac vaccine against symptomatic COVID-19 infection was 46.8% [20]. Another previous study Kaabi et al. demonstrated an efficacy of 78.1–72.8% in reducing the risk of symptomatic COVID-19 after the primary vaccination with inactivated COVID-19 vaccines [21].

This study demonstrated that the administration of the COVID-19 booster dose significantly increased IgG sRBD antibody titres in ESKD patients undergoing routine haemodialysis. A previous study by Patyna et al. also revealed a significant increase in antibody titres to 4,560 BAU/mL (646.7 – 7,272.5) (*p* < 0.001) among haemodialysis patients at 1 month after the booster vaccine [22]. Likewise, Shashar et al. also demonstrated that the IgG sRBD antibody titres increased significantly to 16,336.8 ± 15,397.3 AU/mL (*p* < 0.0001) in patients undergoing routine haemodialysis [12]. Bruminhent et al. evaluated the immunogenicity of a booster dose of ChAdOx1 nCoV-19 after primary vaccination of inactivated SARS-CoV-2 vaccines among dialysis patients. The study also showed that among haemodialysis patients, the anti-RBD IgG level and % neutralization by sVNT increased significantly from 85.3 BAU/mL (IQR 1106–3762.3) and 47.9% (IQR 13.5–85.4), respectively, to 1740.9 BAU/mL (1106–3762.3) and 99.4% (IQR 98.8–99.7), after the booster dose [23]. Another study by Cheng et al. also revealed a similar finding i.e., a significant increase of anti-RBD IgG after a heterologous booster dose with mRNA vaccine (AZ followed by Moderna) among haemodialysis patients. The anti-RBD IgG level increased from 1342.0 ± 1894.0 AU/mL to 22,011 ± 1016 AU/mL [24].

In addition, Quiroga et al. also revealed that a booster dose induced a strong humoral response as indicated by a significant increase in antibody titres after booster vaccines (142 [29–1666] IU/mL vs 6021 [1405–10 000] IU/mL] (*p* < 0.001) [25]. Another study by Agur et al. exhibited a significant increase in the S1-RBD antibody titre from 2.15 ± 0.75 to 3.99 ± 0.83 AU/mL among haemodialysis patients who received BNT162b2 as the booster dose [3].

In our study, the subjects with increasing IgG sRBD antibody titres were younger compared to subjects with declining antibody titres. This is consistent with a previous study that demonstrated that age has a significant effect on antibody levels after vaccination [26]. There is an inverse correlation between age and IgG levels in the dialysis group (*p* = 0.004). In the dialysis group, older age is associated with a lower antibody response [26]. Likewise, the same finding has been demonstrated in the general population. There is an inverse correlation between post-vaccination anti-S titre and age, with individuals older than 50 years generating a significantly weaker serological response than those younger than 50 years [19].

Laboratory values of haemoglobin, lymphocyte count, platelet count, BUN, creatinine, ferritin, and albumin were not significantly different between the two groups. This is contrary to the results of a previous study by Agur et al. The study revealed that low albumin levels affect lower antibody responses in booster vaccines. This could be caused by the much smaller number of subjects in our study. The frequencies of comorbidities, including DM, hypertension, anaemia, and joint pain, did not differ significantly between the two groups. This finding is similar to the study by Agur et al., which showed that the proportion of diabetes mellitus did not affect the antibody response [3].

The IgG sRBD antibody titre before the booster dose was significantly lower in subjects with increasing antibody titres after the booster dose (*p* = 0.004). This finding is clinically significant for patients who experience antibody waning after the primary vaccination. We can conclude that for these patients, the administration of a booster dose is beneficial and able to mount an adequate immune response by significantly increasing the IgG sRBD antibody titres. Therefore, the administration of a booster dose is recommended in haemodialysis patients whose antibody levels decline over time after the primary vaccination. Meanwhile, a proportion of patients maintain high antibody titres 8 months after the primary vaccination, indicating good serological persistence and longevity among haemodialysis patients. Similarly, Bensouna et al. demonstrated that subjects with low antibody titre after the second dose underwent a higher increase in antibody titres after the booster dose [27]. This result suggests that booster dose administration is beneficial for the population that mounts a low antibody response after primary dose vaccination.

In the additional analysis, our study also demonstrated that patients who experienced COVID-19 symptoms between the second dose and the booster dose had a significantly greater increase in IgG sRBD titre (ratio of IgG sRBD at V4 compared to V3) than the asymptomatic ones. This finding implies that patients who experienced COVID-19 symptoms and are thus suspected to have been infected with COVID-19 underwent a more robust humoral response. Similarly, a previous study by Karakizlis et al. also demonstrated that haemodialysis patients with COVID-19 past infections sustained higher anti-SARS-CoV-2-spike levels compared to infection-naïve patients after the primary vaccination. However, both groups reached the upper detection limit of 40,000 AU/mL after the booster dose administration [28].

This study showed that 3 subjects (10.71%) did not experience an increase in the NAbs percentage of inhibition, but had an increase in IgG sRBD antibody titres after vaccination. Comparably, Nayak et al. showed that almost half of the study subjects did not induce neutralizing antibodies but experienced an increase in IgG sRBD antibody titres after COVID-19 infection. These conditions may be related to variations in immune responses between different individuals [29].

Regarding the IgG sRBD GMT, our study showed different results from Shashar et al. [12]. In the study by Shashar et al., the IgG sRBD titre after the booster dose reached 16,336.8 AU/mL. Meanwhile, in our study, the IgG sRBD titre increased to 23,050 U/mL after the booster dose. This finding might be caused by different methods of antibody measurement. Our study utilized the Electro Chemiluminescence Immunoassay (ECLIA) (sensitivity 96%, specificity 99.9%), whereas Shashar et al. employed the Chemiluminescent Microparticle Immunoassay (CMIA) (sensitivity 93%, specificity 99.5%) [30].

In this study, the most common AEs after the booster dose include mild pain at the injection site (55.26%), mild fatigue (10.53%), and swelling at the injection site (10.53%). This finding is similar to the BNT162b2 booster vaccine clinical trial that revealed pain at the injection site (12.9%) and fatigue (7.2%) as the most common AEs [31]. No serious or life-threatening adverse events were reported among the study subjects after the booster dose.

The main strength of this study is its cohort prospective design, in which patients are followed-up for four weeks after the booster dose vaccination. Also, in this study, the immunogenicity was evaluated by measuring the neutralizing antibody levels against sRBD with the sVNT method, one of the most reliable methods to estimate the neutralizing antibody levels in serum. The limitation of this study is the relatively small number of samples. In addition, this study only assessed the immunogenicity based on only the humoral responses, not along with the cellular responses. As is known, the cellular immune responses also play crucial roles in protecting against COVID-19 [32]. Moreover, past COVID-19 infections could be confirmed since not all patients underwent RT-PCR. Instead, past COVID-19 infections could only be inferred from patients’ history of symptoms.

## Conclusions

ESKD patients undergoing routine haemodialysis mounted a good antibody response to the COVID-19 BNT162b2 booster dose vaccination with tolerable adverse events.

## Supplementary Information

**Additional file 1.**

**Additional file 2.**

## Data Availability

The datasets supporting the conclusions of this article are included within the article and its additional files.

## Abbreviations

BUN: Blood Urea Nitrogen
CKD: Chronic Kidney Disease
CMIA: Chemiluminescent Microparticle Immunoassay
ECLIA: Electro Chemiluminescence Immunoassay
EKSD: End-Stage Kidney Disease
FDA: Food and Drug Administration
GMFR: Geometric Mean Fold Rise
GMT: Geometric Mean Titre
HD: Haemodialysis
IgG: Immunoglobulin G
IQR: Inter Quartile Range
NAbs: Neutralizing Antibody
RT-PCR: *Real Time* Polymerase Chain Reaction
sVNT: Surrogate Virus Neutralization Test
