# Supplementary material for: Immunogenicity and safety of COVID-19 BNT162b2 booster vaccine in end-stage kidney disease patients receiving haemodialysis in Yogyakarta, Indonesia: a cohort prospective study
Source: BMC Nephrol. 2023 May 30;24:151. doi: 10.1186/s12882-023-03218-x (PMC10226875; doi:10.1186/s12882-023-03218-x)
Supplement: Supplementary file 1 — Additional file 1. [file 12882_2023_3218_MOESM1_ESM.docx]

**Additional File 1**

**Supplementary Table 1.** Adverse events (AE) based on the onset.

| **Adverse events** | **N** | **%** |  |
| --- | --- | --- | --- |
|  |  |  |  |
| **30 minutes** |  |  |  |
| ***Solicited* AE** |  |  |  |
| Subjects with at least one solicited AE | 5 | 13,16 |  |
| The total number of solicited AE | 5 |  |  |
| Total number of severe solicited AE / ≥ Grade 3 | 0 | 0 |  |
| ***Unsolicited* AE** |  |  |  |
| Subjects with at least one unsolicited AE | 0 | 0 |  |
| The total number of unsolicited AE | 0 | 0 |  |
| Total number of severe unsolicited AE / ≥ Grade 3 | 0 | 0 |  |
| **Serious AE** |  |  |  |
| Subjects with at least one serious AE |  |  |  |
| The total amount of serious AE | 0 | 0 |  |
| **Day 1** |  |  |  |
| ***Solicited* AE** |  |  |  |
| Subjects with at least one solicited AE | 19 | 50 |  |
| The total number of solicited AE | 26 |  |  |
| Total number of severe solicited AE / ≥ Grade 3 | 0 | 0 |  |
| ***Unsolicited* AE** |  |  |  |
| Subjects with at least one unsolicited AE | 0 | 0 |  |
| The total number of unsolicited AE | 0 | 0 |  |
| Total number of severe unsolicited AE / ≥ Grade 3 | 0 | 0 |  |
| **Serious AE** |  |  |  |
| Subjects with at least one serious AE |  |  |  |
| The total amount of serious AE | 0 | 0 |  |
| **Day 2 – day 7** |  |  |  |
| ***Solicited* AE** |  |  |  |
| Subjects with at least one solicited AE | 12 | 31,58 |  |
| The total number of solicited AE | 17 |  |  |
| Total number of severe solicited AE / ≥ Grade 3 | 1 | 5,88 |  |
| ***Unsolicited* AE** |  |  |  |
| Subjects with at least one unsolicited AE | 1 | 2,63 |  |
| The total number of unsolicited AE | 1 |  |  |
| Total number of severe unsolicited AE / ≥ Grade 3 | 1 | 100 |  |
| **Serious AE** |  |  |  |
| Subjects with at least one serious AE |  |  |  |
| The total amount of serious AE | 0 | 0 |  |
| **Day 8 – day 14** |  |  |  |
| ***Solicited* AE** |  |  |  |
| Subjects with at least one solicited AE | 0 | 0 |  |
| The total number of solicited AE | 0 | 0 |  |
| Total number of severe solicited AE / ≥ Grade 3 | 0 | 0 |  |
| ***Unsolicited* AE** |  |  |  |
| Subjects with at least one unsolicited AE | 3 | 7,89 |  |
| The total number of unsolicited AE | 3 |  |  |
| Total number of severe unsolicited AE / ≥ Grade 3 | 0 | 0 |  |
| **Serious AE** |  |  |  |
| Subjects with at least one serious AE | 0 | 0 |  |
| The total amount of serious AE | 0 | 0 |  |

*AE* adverse events
