# Supplementary material for: Immunogenicity and safety of COVID-19 BNT162b2 booster vaccine in end-stage kidney disease patients receiving haemodialysis in Yogyakarta, Indonesia: a cohort prospective study
Source: BMC Nephrol. 2023 May 30;24:151. doi: 10.1186/s12882-023-03218-x (PMC10226875; doi:10.1186/s12882-023-03218-x)
Supplement: Supplementary file 2 — Additional file 2. [file 12882_2023_3218_MOESM2_ESM.docx]

**Additional File 2.**

**Supplementary Table 2.** Solicited adverse events grouped by intensity.

| **Intensity** | n | % of |
| --- | --- | --- |
|  | subject | subject |
| **Local** | | |
| Pain |  |  |
| Grade 1 / Mild | 21 | 55,26 |
| Grade 2 / Moderate | 0 | 0 |
| Grade 3 / Severe | 0 | 0 |
| Grade 4 / Life-threatening | 0 | 0 |
| Swelling |  |  |
| Grade 1 / Mild | 4 | 10,53 |
| Grade 2 / Moderate | 0 | 0 |
| Grade 3 / Severe | 0 | 0 |
| Grade 4 / Life-threatening | 0 | 0 |
| Redness |  |  |
| Grade 1 / Mild | 0 | 0 |
| Grade 2 / Moderate | 0 | 0 |
| Grade 3 / Severe | 0 | 0 |
| Grade 4 / Life-threatening | 0 | 0 |
| **Systemic** | | |
| Fever |  |  |
| Grade 1 / Mild | 2 | 5,26 |
| Grade 2 / Moderate | 0 | 0 |
| Grade 3 / Severe | 0 | 0 |
| Grade 4 / Life-threatening | 0 | 0 |
| Fatigue |  |  |
| Grade 1 / Mild | 4 | 10,53 |
| Grade 2 / Moderate | 0 | 0 |
| Grade 3 / Severe | 1 | 2,63 |
| Grade 4 / Life-threatening | 0 | 0 |
| Cough |  |  |
| Grade 1 / Mild | 0 | 0 |
| Grade 2 / Moderate | 0 | 0 |
| Grade 3 / Severe | 0 | 0 |
| Grade 4 / Life-threatening | 0 | 0 |
| Nausea |  |  |
| Grade 1 / Mild | 0 | 0 |
| Grade 2 / Moderate | 0 | 0 |
| Grade 3 / Severe | 0 | 0 |
| Grade 4 / Life-threatening | 0 | 0 |
| Vomitus |  |  |
| Grade 1 / Mild | 1 | 2,63 |
| Grade 2 / Moderate | 0 | 0 |
| Grade 3 / Severe | 0 | 0 |
| Grade 4 / Life-threatening | 0 | 0 |
| Diarrhea |  |  |
| Grade 1 / Mild | 0 | 0 |
| Grade 2 / Moderate | 0 | 0 |
| Grade 3 / Severe | 0 | 0 |
| Grade 4 / Life-threatening | 0 | 0 |
| Headache |  |  |
| Grade 1 / Mild | 1 | 2,63 |
| Grade 2 / Moderate | 0 | 0 |
| Grade 3 / Severe | 0 | 0 |
| Grade 4 / Life-threatening | 0 | 0 |
| Muscle pain |  |  |
| Grade 1 / Mild | 1 | 2,63 |
| Grade 2 / Moderate | 0 | 0 |
| Grade 3 / Severe | 0 | 0 |
| Grade 4 / Life-threatening | 0 | 0 |
| Hypersensitivity |  |  |
| Grade 1 / Mild | 0 | 0 |
| Grade 2 / Moderate | 0 | 0 |
| Grade 3 / Severe | 0 | 0 |
| Grade 4 / Life-threatening | 0 | 0 |
| Other digestive tract symptoms |  |  |
| Grade 1 / Mild | 0 | 0 |
| Grade 2 / Moderate | 0 | 0 |
| Grade 3 / Severe | 0 | 0 |
| Grade 4 / Life-threatening | 0 | 0 |
